# Reliability of the Moover® 3D Inertial Motion Sensor in Greek Patients With Chronic Neck Pain in a Primary Care Urban Setting

**DOI:** 10.7759/cureus.66336

**Published:** 2024-08-06

**Authors:** Charalampos Skordis, Andreas Mavrogenis, George Georgoudis

**Affiliations:** 1 Department of Physiotherapy and Musculoskeletal Physiotherapy Research Laboratory, University of West Attica (UNIWA), Athens, GRC; 2 1st Orthopedic Department, Attikon University General Hospital, National and Kapodistrian University of Athens, Athens, GRC

**Keywords:** wearable sensors, chronic neck pain, inter-rater reliability, intra-rater reliability, 3d moover, inertial sensors, cervical range of motion (rom)

## Abstract

Introduction

Neck pain has a high lifetime prevalence and represents a significant health issue. Reduced active cervical range of motion (ACROM) has been found in neck pain patients. Inertial sensor technology can provide objective measurements to assess the impaired ACROM.

Purpose

Primarily, this study investigated the inter- and intra-rater reliability of the Moover® three-dimensional (3D) inertial motion sensor (Sensor Medica, Rome, Italy) in Greek patients with non-specific chronic neck pain. Secondly, the intra-rater reliability of the Neck Disability Index (NDI) was also assessed.

Methods

Fifty patients (18 males and 32 females) suffering from non-specific chronic neck pain participated in this study. Two physiotherapists measured separately each participant’s ACROM in three planes, within a 48-hour period. The participants’ position and the sequence and direction of the three cervical movements (cervical rotation, lateral flexion, and flexion-extension) were standardized.

Results

The inter-rater reliability intraclass correlation coefficient (ICC) values were good to excellent ranging from 0.77 to 0.95 for the first measurement and 0.85 to 0.95 for the second (p < 0.001). The intra-rater reliability ICC values were moderate to excellent ranging from 0.74 to 0.92 for the first rater and good to excellent ranging from 0.83 to 0.94 for the secondrater (p < 0.001). Intra-rater reliability of the overall NDI was indicated as good, and ICC was 0.80 (95%CI: 0.65-0.89; p < 0.001). ICC values for all sections were significant and ranged from 0.40 to 0.88.

Conclusion

This study showed the reliability of the Moover 3D inertial sensor for ACROM measurement in Greek patients with chronic neck pain. The NDI scale also showed good intra-rater reliability in the same sample. Both intra- and inter-rater reliability of the Moover 3D were proven to be acceptable over a 48-hour period. The specific sensor might have a potential application in a clinical setting.

## Introduction

Globally, neck pain is still a significant public health burden [1], with an estimated lifetime prevalence range of 14-71% [2]. The active cervical range of motion (ACROM) measurement is a clinical objective tool that can be an index of neck pain pathology and it can also be used to evaluate the process of a therapeutic plan [3,4]. In people with neck pain, reduced smoothness of movement and a total reduction of ACROM in all planes have been found [5].

Many instruments have been researched to measure ACROM including various types of goniometers and inclinometers, tape measurements, visual estimation, mobile applications, electromagnetic trackers, ultrasonic techniques, and optical motion capture [6-8]. Inertial wearable sensors are tools that provide valid and reliable quantitative assessments of physical impairment through ACROM in three planes [8-10].

The number of sensors applied and device placement could influence the reliability of the measurements [10]. Ideally, a single sensor device is desired [3]. Most studies recommend the use of two sensors not only for higher precision but also for the elimination of undesired compensation body movements or coupled movements mainly in the thoracic region [3,10-12].

Among the most reliable locations for sensor placement is one sensor on the forehead and one on the T4 vertebra [13]. Three studies using two sensors placed the first sensor on the forehead and the second on the T4 vertebra posteriorly [3,8], or anteriorly on the thorax at the level of the sternal manubrium [9]. In a study by Raya et al., excellent inter-rater (ICC = 0.93) and test-retest reliability (ICC > 0.90) were reported in 27 asymptomatic adults [3]. High intra-rater reliability for both raters (ICC > 0.90) and good inter-rater reliability (ICC > 0.75) were found in 20 asymptomatic adults in a study by Anoro-Hervera et al. [8]. In another study, by Elizagaray-García et al., good intra-rater values were obtained for the first rater (ICC > 0.80) and the second rater (ICC > 0.84), while the inter-rater values were greater than 0.75 in 20 adults with chronic primary headache [9].

Only one study evaluated the reliability of a single sensor attached to the forehead in an asymptomatic sample, with test-retest reliability to be moderate to good for younger adults (ICC: 0.54-0.82) and poor when accounting for the difference between the start and ending position named Zero Point [14]. In the same study, older adults showed moderate to excellent agreement ranging between 0.65-0.92.

No study was found to have investigated the reliability of the Moover® three-dimensional (3D) inertial motion sensor (Sensor Medica, Rome, Italy) utilizing a single sensor attached to the forehead of patients with chronic neck pain. Only one study has been found to use the same device and placement on the forehead. This study has examined the immediate and short-term effect of elastic taping application to the cervical area on ACROM and self-perceived pain in video terminal operator workers with chronic musculoskeletal cervical pain. The accuracy and validity of the Moover 3D have been assessed in a preliminary comparison with an optoelectronic system by Russo et al. (which the authors declare to serve as the gold standard) in 19 subjects, with no significant differences in ACROM measurement between the two instruments [15]. However, the reliability assessment was not the purpose of their study. Therefore, there is a lack of research evaluating the intra- and inter-rater reliability of the single inertial motion sensor of the 3D Moover in patients with chronic neck pain.

Taking into consideration the lack of relevant research into the evaluation of the intra and inter-rater reliability of the single inertial motion sensor of the Moover 3D in patients with chronic neck pain, the current study aimed at shedding light on this overlooked area of interest. More specifically, the primary purpose of our study was to evaluate the reliability of the Moover, a 3D single inertial motion sensor, in Greek patients with chronic neck pain who have not undergone any treatment. The secondary purpose was to assess the intra-rater reliability of the Neck Disability Index (NDI).

## Materials and methods

Study design

An intra- and inter-rater design was selected for this study. Additionally, the intra-rater reliability of the NDI was also assessed. The study was conducted in the “Mikis Theodorakis” Multipurpose Center for Culture, Sports and Social Activities of Ilion in Athens, Greece, in collaboration with the Musculoskeletal Physiotherapy Research Lab of the University of West Attica (UNIWA) in Athens from January 2023 to August 2023. All measurements were taken by two experienced physiotherapists, in the use of the Moover device. Written informed consent was obtained from all subjects. The study was approved by the Research Ethics and Ethics Committee of the University of West Attica (UNIWA), with protocol number: 103276/18-12-2020.

Participants

Fifty patients suffering from non-specific chronic (≥ 3 months) neck pain, 18 men (36%) and 32 women (64%), most of whom were in the age group of 50-59 years (40%), participated in the study. The exclusion criteria were neck pain related to neurological disorders, systematic inflammatory disease, rheumatic diseases, another known pathological cause, previous surgery, or any kind of trauma at least two years ago. Patients receiving other treatments during the study were also excluded.

The power analysis revealed a sample of 50 participants, for finding an ICC of at least 0.8 significant, necessary to provide a power of 80% at a significance level of 0.05 [16,17].

Instrumentation

The measurement instrument for the cervical range of motion was the 3D Moover. The device is a small portable wireless inertial sensor connected by Bluetooth 3.0 to the installed Freestep application software on the computer. The sensor records mechanical cervical spine motion. It allows goniometric evaluation, acceleration, and rotation and converts them into an electrical signal. The accelerometer can record range of motion (ROM) and acceleration values in three axes [18]. Through a software application, all recorded data are converted into diagrams, and an automatic report is displayed.

Outcome measure

Neck Disability Index (NDI)

The NDI which is an alteration of the Oswestry Low Back Pain questionnaire originally developed in 1991 [11,19], is a valid worldwide popular tool [19]. It is a self-rated questionnaire that investigates cervical disability within subjects’ lives and consists of a total of 10 items. Each section has six answer choices which can be scored from 0 to 5, therefore the total range is 0-50. The higher the score, the greater the level of neck disability. In this research study, the Greek-modified version is used, which is valid, reliable, and appropriate to use in neck pain patients [20,21].

Procedure

Patients were measured twice within a 48-hour period [9]. Before the first measurement, the patients were informed about the study and written consent was provided. Then, subjects completed all demographic questions and the NDI.

**Figure 1:**
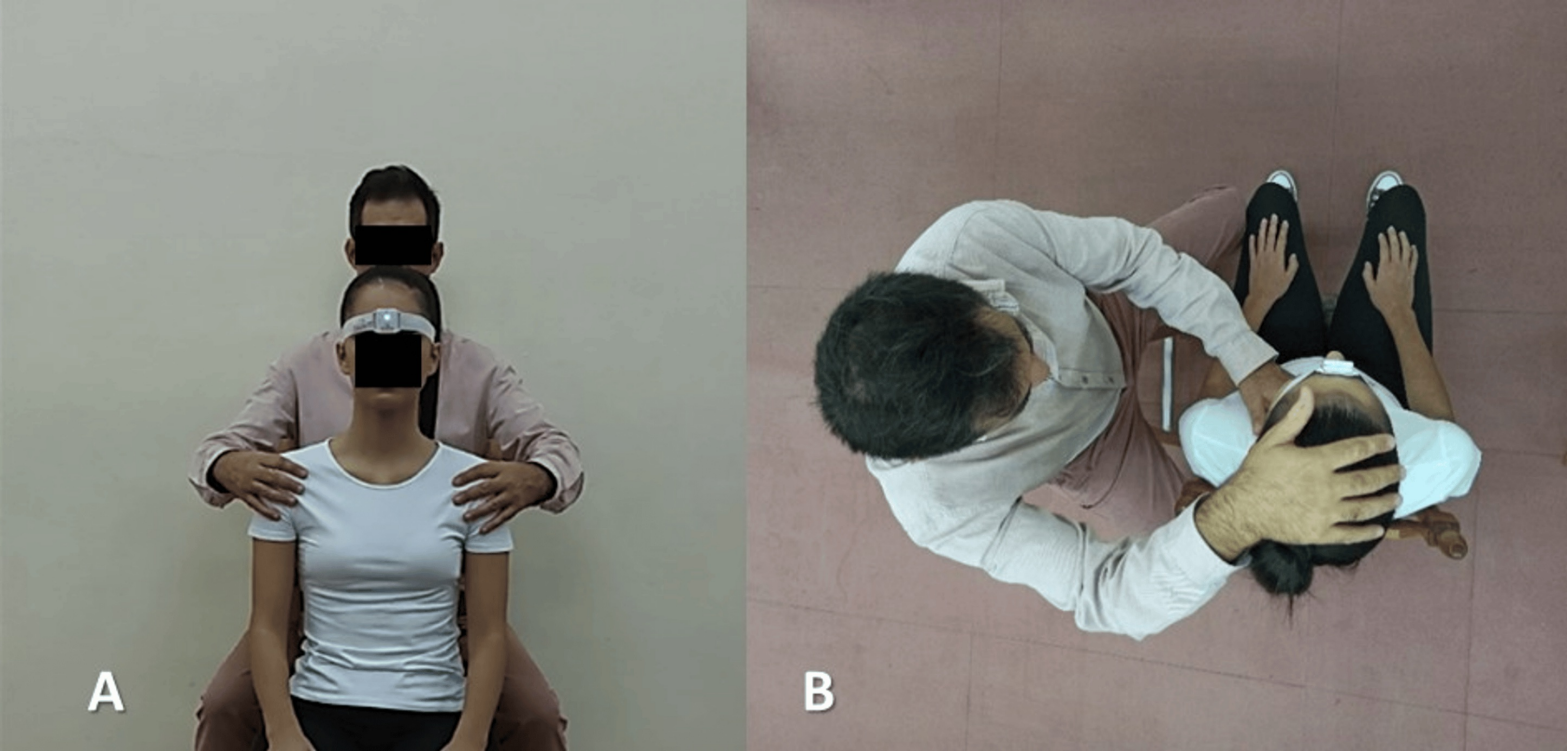
Sensor placement and calibration. (A) Sensor placement on forehead; (B) Sensor calibration at the neutral or starting position.

Afterward, the device was positioned in the center of the forehead (Figure 1A) on the frontal bone at the level of the glabella, which is above the nasal bridge, and it was fastened around the head with the assistance of a strap [18].

A standardized neutral body position or starting position was established as a reference point to diminish variability in measurements [22]. For this reason, each participant was seated on a chair facing a wall, with their head in a neutral position maintaining a horizontal gaze [14]. Their back formed a 90-degree angle perpendicular to the hip joint, with the shoulders relaxed, the arms alongside the body, and hands rested on the thighs. The sacrum and scapula were in constant contact with the backrest of the chair. The knees were placed at a 90-degree angle and the feet were flat on the floor [3,9,18].

After the seated position was secured, a masking tape was placed in front of each subject's level of gaze, in order to assist their understanding of the cervical motion planes and to avoid compensation motions. Specifically, during the lateral flexions, the subject used the center of the tape as a reference point to avoid rotations in their effort to achieve maximum ROM, while during rotations they used it as a guide to refrain from compensating motions in other planes or axes [3].

Before the cervical ROM measurement, the individual was instructed on the proper execution of the testing cervical movement while seated in the neutral position. In order to reassure the participant’s understanding that the testing motions and coupled motions were restricted, a short time for practice was given if needed [4] under the guidance of the rater. Then the rater calibrated the device in the above-mentioned neutral body position (Figure 1B) as given below.

Eyes and jaw were aligned at the horizontal level of the floor with the assistance of the tape, nose, mouth, and chin were vertically aligned, and the sensor was calibrated by the rater [3,22]. Calibration was applied first for each subject prior to each of the three motion measurements separately, followed by the measurement of the selected movement by the rater.

**Figure 2:**
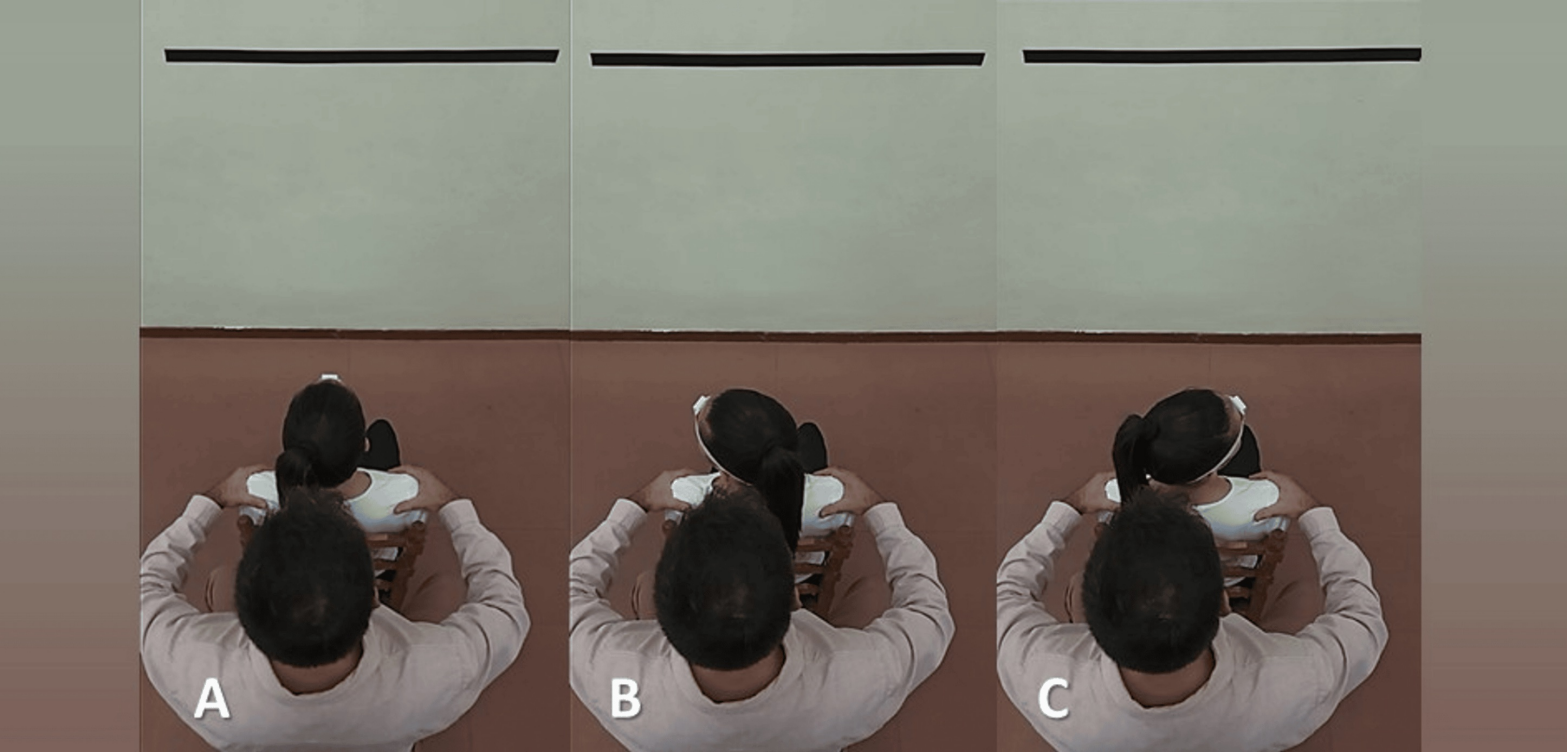
Cervical rotation. (A) Neutral or starting position; (B) Maximum left rotation; (C) Maximum right rotation

**Figure 3:**
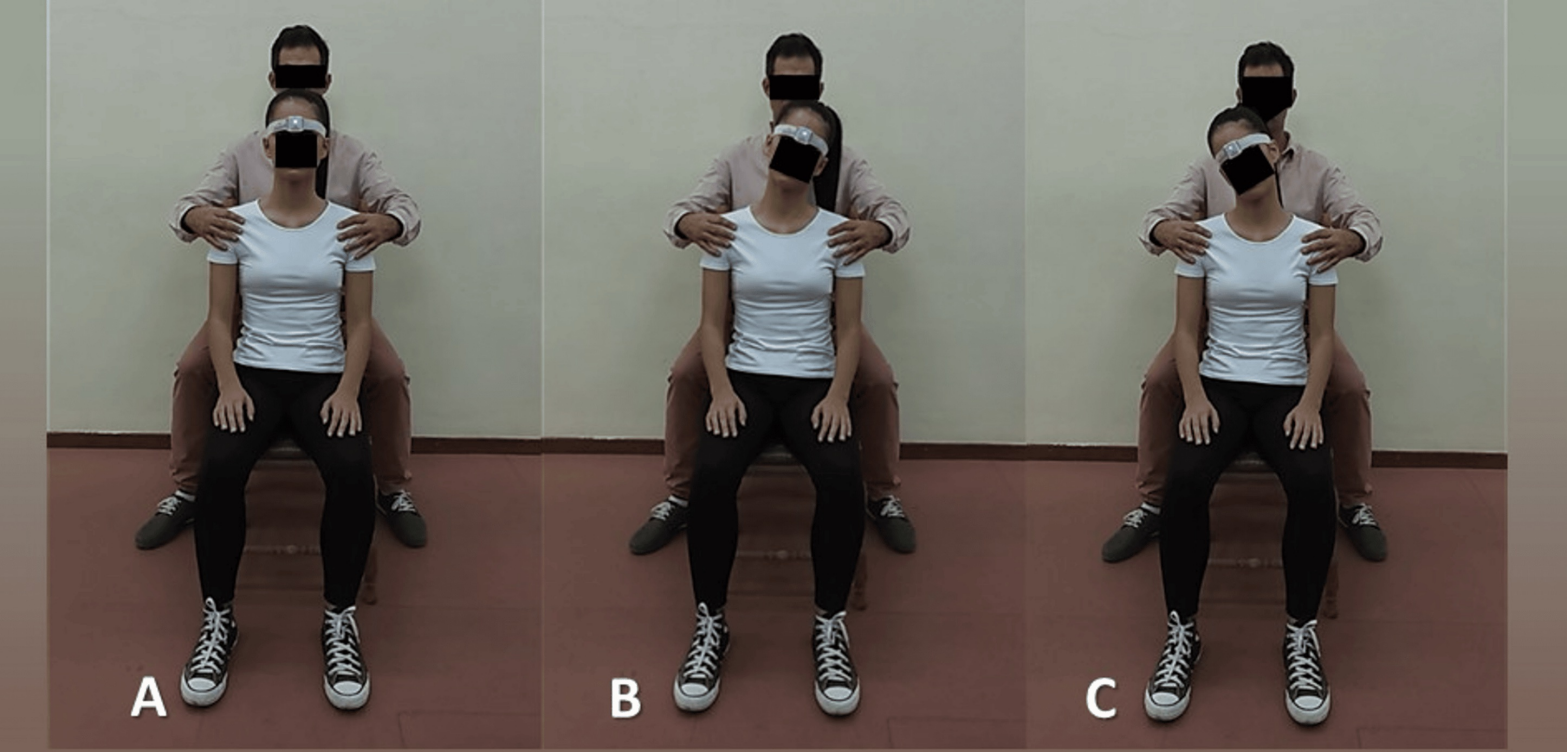
Cervical lateral flexion. (A) Neutral or starting position; (B) Maximum left lateral flexion; (C) Maximum right lateral flexion

**Figure 4:**
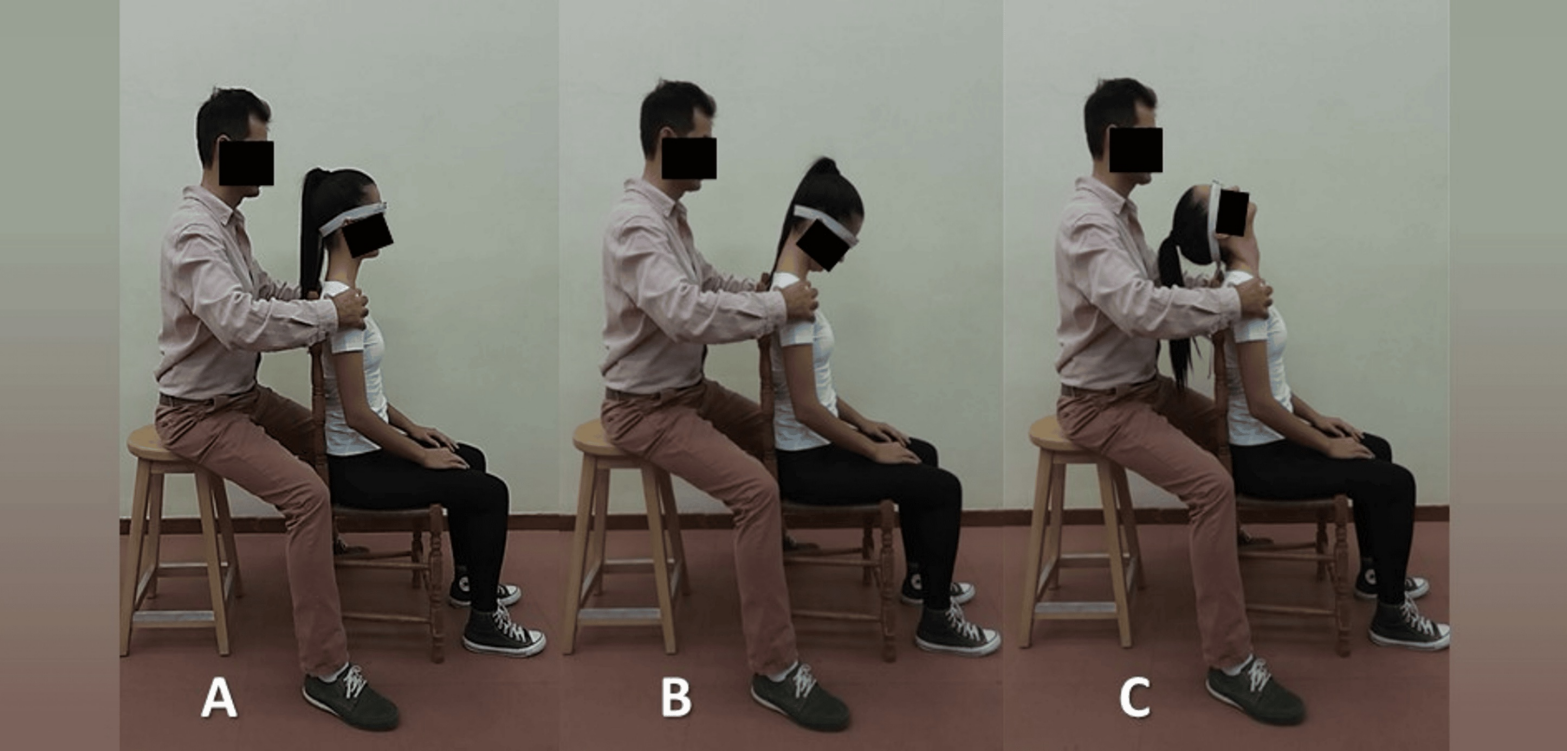
Cervical flexion-extension. (A) Neutral or starting position; (B) Maximum flexion; (C) Maximum extension

The order and direction of the three cervical movements recorded were determined by the software application and instructed as follows: First, in total three sequential left-right rotation motions were performed starting from the neutral position and going to the maximal left rotation position first (Figure 2). Second, in total three sequential left-right lateral flexion motions were performed starting from the neutral position and going to the maximal left lateral flexion position first (Figure 3). Third, in total three sequential flexion-extension motions were performed starting from the neutral position and going to the maximal flexion position first (Figure 4).

Each movement was performed separately from each other. Patients were encouraged to perform the testing movement in maximal full-ROM in a smooth flow pausing briefly in the end range. Feedback was given to the patient when needed during the measurements. If the subject did not follow the instructions given, the measurement was not saved and then the whole process was repeated.

To avoid measurement errors due to trunk compensating movements, active stabilization of the trunk and the shoulders was applied manually by the rater’s hands placed over the distal clavicle and acromion region [22] while sitting directly behind the subject and passive stabilization was provided by the backrest of the chair (Figures 2-4).

When the measurements were successfully completed, a five-minute break was offered to the patient to minimize the fatigue effect and then a second rater repeated the same three motions [10] following the same protocol. Finally, the whole process was repeated in 48 hours in the same manner [9]. Both raters were blind to participants’ measurements and recorded data [22]. The second rater was blind to previous measurement results and both raters were blinded to the subject’s clinical information and the data of measurement values recorded [23].

Statistical analysis

For the description of quantitative variables, mean (standard deviation (SD)) or median (interquartile range (IQR)) were used. For describing categorical variables, absolute and relative frequencies were used. Intra- and inter-rater reliability were checked via intraclass correlation coefficients (ICCs) and their 95% confidence Intervals (CI). The ICC is a value between 0 and 1, where values below 0.50 indicate poor reliability, between 0.50 and 0.75 moderate reliability, and between 0.75 and 0.90 good reliability, whereas any value above 0.90 indicates excellent reliability [24]. ROM and NDI values were tested for normality using the Kolmogorov-Smirnov criterion. Since the normality assumption was not satisfied, non-parametric tests were used. Scores in NDI were compared between T1 and T2 using the Wilcoxon signed-rank test. Spearman correlation coefficients (rho) were used to explore the association of two continuous variables. All reported p-values are two-tailed. Statistical significance was set at p < 0.05 and analyses were conducted using IBM SPSS Statistics for Windows, Version 22.0 (Released 2013; IBM Corp., Armonk, New York, United States).

## Results

Fifty participants were included in the study. Their characteristics are presented in Table 1. Most participants were 50-59 years old (40.0%) and were overweight (56%). Also, 32% of the sample were university alumni, 42% were employees in the public sector, and 72% were married. Moreover, 70% of the participants had experienced symptoms for at least 24 months, 58% were under medication, and 64% suffered from another disease.

**Table 1:** Sample characteristics. Values are presented as n (%) except for BMI which has been presented as mean±SD, as indicated

Characteristics		n (%)
Sex		
	Male	18 (36)
	Female	32 (64)
Age (years)		
	30-39	4 (8)
	40-49	13 (26)
	50-59	20 (40)
	60-70	13 (26)
BMI (kg/m^2^), mean (SD)		26.9±4.1
BMI levels		
	Normal	13 (26)
	Overweight	28 (56)
	Obese	9 (18)
Educational level complete		
	Primary	3 (6)
	Secondary	15 (30)
	University	16 (32)
	2-year college	8 (16)
	MSc/ PhD	8 (16)
Work status		
	Unemployed	6 (12)
	Employee in public sector	21 (42)
	Freelancer	3 (6)
	Employee in private sector	11 (22)
	Pensioner	9 (18)
Family status		
	Unmarried	6 (12)
	Married	36 (72)
	Divorced	6 (12)
	Widowed	2 (4)
Symptom duration (months)		
	3-6	6 (12)
	6-12	2 (4)
	12-24	7 (14)
	>24	35 (70)
Medication		29 (58)
Other disease		32 (64)

Intra- and inter-rater reliability results for cervical ROM are presented in Table 2. The inter-rater reliability indexes ranged from 0.77 to 0.95 for the first measurement and from 0.85 to 0.95 for the second measurement and all were significant at p < 0.001. The intra-rater reliability indexes ranged from 0.74 to 0.92 for the first rater and from 0.83 to 0.94 for the second rater and all were significant at p < 0.001. Thus, the cervical ROM showed good to excellent reliability in most of the cases. 

**Table 2:** Intra- and inter-rater reliability results for cervical range of motion. ICC: intra-class correlation coefficient

	1^st^ Measurement (T1)		2^nd^ Measurement (T2)		ICC (95% CI)		ICC (95% CI)	
	Rater 1	Rater 2	Rater 1	Rater 2	Inter-rater reliability		intra-rater reliability	
Motion (°)	Mean (SD)	Mean (SD)	Mean (SD)	Mean (SD)	1^st ^measurement (T1)	2^nd^ measurement (T2)	Rater 1	Rater 2
Rotation maximum								
Left	60.3 (9.7)	62.6 (9.3)	61.8 (11.1)	62.4 (10.5)	0.92 (0.86 ─ 0.96)	0.90 (0.83 ─ 0.94)	0.81 (0.67 ─ 0.89)	0.87 (0.77 ─ 0.92)
Right	60.0 (9.8)	59.9 (9.4)	60.1 (10.1)	60.6 (9.1)	0.81 (0.66 ─ 0.89)	0.87 (0.77 ─ 0.93)	0.83 (0.70 ─ 0.90)	0.94 (0.89 ─ 0.96)
Rotation average								
Left	58.8 (10)	61.1 (10.0)	60.2 (10.8)	61.1 (10.9)	0.89 (0.80 ─ 0.93)	0.85 (0.73 ─ 0.91)	0.75 (0.56 ─ 0.86)	0.86 (0.76 ─ 0.92)
Right	57.2 (9.8)	57.5 (10.1)	58.4 (10.2)	58.8 (10.0)	0.77 (0.59 ─ 0.87)	0.87 (0.77 ─ 0.93)	0.74 (0.55 ─ 0.85)	0.83 (0.70 ─ 0.90)
Lateral flexion maximum								
Left	30.7 (7.4)	32.4 (7.1)	29.9 (8.3)	31.3 (8.2)	0.90 (0.83 ─ 0.94)	0.93 (0.88 ─ 0.96)	0.87 (0.78 ─ 0.93)	0.92 (0.86 ─ 0.96)
Right	30.7 (7.6)	31.6 (8.0)	30.3 (8.4)	31.1 (7.6)	0.95 (0.90 ─ 0.97)	0.95 (0.90 ─ 0.97)	0.91 (0.84 ─ 0.95)	0.88 (0.79 ─ 0.93)
Lateral flexion average								
Left	29.8 (7.3)	31.7 (7.2)	29.3 (8.2)	30.6 (8.1)	0.90 (0.82 ─ 0.94)	0.94 (0.89 ─ 0.96)	0.87 (0.77 ─ 0.92)	0.93 (0.88 ─ 0.96)
Right	29.8 (7.5)	30.8 (7.9)	29.5 (8.2)	30.3 (7.5)	0.95 (0.91 ─ 0.97)	0.95 (0.91 ─ 0.97)	0.91 (0.84 ─ 0.95)	0.89 (0.81 ─ 0.94)
Flexion maximum	52.6 (10.5)	51.4 (10.8)	50.6 (12.5)	49.1 (11.2)	0.86 (0.75 ─ 0.92)	0.91 (0.84 ─ 0.95)	0.77 (0.59 ─ 0.87)	0.89 (0.81 ─ 0.94)
Extension maximum	59.3 (12.7)	57.1 (11.9)	56.0 (11.9)	55.8 (12.3)	0.89 (0.81 ─ 0.94)	0.93 (0.87 ─ 0.96)	0.92 (0.85 ─ 0.95)	0.92 (0.86 ─ 0.95)
Flexion average	51.3 (10.5)	50.1 (10.9)	49.0 (12.7)	48.0 (11.2)	0.84 (0.71 ─ 0.91)	0.90 (0.83 ─ 0.94)	0.75 (0.56 ─ 0.86)	0.89 (0.81 ─ 0.94)
Extension average	57.5 (12.4)	55.5 (11.6)	54.8 (11.8)	54.6 (12.4)	0.89 (0.81 ─ 0.94)	0.93 (0.88 ─ 0.96)	0.92 (0.85 ─ 0.95)	0.92 (0.86 ─ 0.95)

 Intra-rater reliability results for NDI are presented in Table 3. ICCs for all sections were significant and ranged from 0.40 to 0.88. For the overall NDI, ICC was 0.80 (95%CI: 0.65-0.89; p < 0.001), indicating good reliability.

**Table 3:** Intra-rater reliability results for Neck Disability Index. ^1^Intra-class Correlation Coefficient (95% Confidence Interval) for intra-rater reliability, ^2 ^Wilcoxon signed-rank test. ^*^ p ≤ 0.05, ^**^ p ≤ 0.01, ^***^ p ≤ 0.001.

	1^st^ Measurement (T1)	2^nd^ Measurement (T2)		
	Median (IQR)	Median (IQR)	ICC^1^ (95% CI)	Ρ^2^
Section 1 - Pain Intensity (0-5)	1 (1 ─ 2)	1 (1 ─ 2)	0.55 (0.21 ─ 0.75)	0.003^**^
Section 2 - Personal Care (0-5)	0 (0 ─ 1)	0 (0 ─ 1)	0.40 (0.00 ─ 0.66)	0.041^*^
Section 3 - Lifting (0-5)	1 (0 ─ 2)	1 (1 ─ 1)	0.84 (0.72 ─ 0.91)	< 0.001^***^
Section 4 - Reading (0-5)	2 (1 ─ 2)	1 (1 ─ 2)	0.74 (0.55 ─ 0.85)	< 0.001^***^
Section 5 - Headache (0-5)	2 (1 ─ 3)	2 (1 ─ 3)	0.78 (0.60 ─ 0.87)	< 0.001^***^
Section 6 - Concentration (0-5)	1 (0 ─ 2)	1 (0 ─ 2)	0.88 (0.78 ─ 0.93)	< 0.001^***^
Section 7 - Work (0-5)	1 (0 ─ 2)	1 (0 ─ 1)	0.75 (0.57 ─ 0.86)	< 0.001^***^
Section 8 - Driving (0-5)	1 (0 ─ 2)	1 (1 ─ 1)	0.76 (0.57 ─ 0.87)	< 0.001^***^
Section 9 - Sleeping (0-5)	1 (1 ─ 2)	1 (1 ─ 1)	0.71 (0.49 ─ 0.84)	< 0.001^***^
Section 10 - Recreation (0-5)	1 (1 ─ 2)	1 (1 ─ 2)	0.61 (0.32 ─ 0.78)	0.001^***^
	Mean (SD)	Mean (SD)		
Neck disability index	12.7 (5.7)	11 (5.5)	0.80 (0.65 ─ 0.89)	< 0.001^***^

The association of the cervical ROM with NDI is presented in Table 4. Greater NDI score was significantly associated with greater extension maximum (rho = 0.40; p = 0.004) and extension average (rho = 0.36; p = 0.011).

**Table 4:** Spearman correlation coefficients (rho) of cervical range of motion with Neck Disability Index (1st measurement). * p ≤ 0.05, ** p ≤ 0.01, *** p ≤ 0.001.

Cervical range of motion		Neck Disability Index score
Rotation maximum		
Left	rho	0.12
	P	0.414
Right	rho	0.00
	P	0.973
Rotation average		
Left	rho	0.05
	P	0.749
Right	rho	0.08
	P	0.585
Lateral flexion maximum		
Left	rho	0.24
	P	0.091
Right	rho	0.17
	P	0.238
Lateral flexion average		
Left	rho	0.24
	P	0.096
Right	rho	0.20
	P	0.159
Flexion maximum	rho	-0.09
	P	0.515
Extension maximum	rho	0.40
	P	0.004^**^
Flexion average	rho	-0.08
	P	0.560
Extension average	rho	0.36
	P	0.011^*^

## Discussion

This study aimed to investigate the intra- and inter-rater reliability of the 3D Moover device in Greek patients suffering from chronic neck pain in an urban setting. Full ROM movements was chosen as they have shown excellent test-retest reliability compared to half movements because the variability caused by a drift in the neutral position is eliminated [3]. The selection of the 48-hour interval between the two measurement sessions was made for reasons of easier patient recruitment and minimizing the effect of possible external factors (sample habits and activities) on measurements [21].

To the best of our knowledge, no study has investigated the reliability of the 3D Moover in chronic neck pain patients. One study only examined the accuracy and validity of the 3D Moover, but not the reliability [15].

Our inter-rater reliability (ICC) was shown to be good to excellent ranging from 0.77 to 0.95 for the first measurement and 0.85 to 0.95 for the second. Subsequently, the intra-rater reliability is shown to be moderate to excellent ranging from 0.74 to 0.92 for the first rater and good to excellent ranging from 0.83 to 0.94 for the second rater respectively.

The ICC values reported in this study were similar to three studies that assessed the inter- and intra-rater reliability using two inertial sensors [3,8,9]. As the literature suggests, the application of two sensors retrieved some higher values in these studies, but the values in the current study were similar and showed that a single sensor could produce similar results to the two sensors setup, producing reliable results for the ACROM measurement.

The study that included the single sensor on the forehead used the same three movements in the same order as our study but with poorer results [14]. Except for the lumbar support, any other form of stabilization of the thoracic segment during the execution of the movements was not mentioned in their study, thus increasing the risk of coupled movements. An advantage of the current study was the stabilization method provided which was both active and passive yielding higher reliability values compared to former published studies.

Since neck pain intensity can limit the ACROM and functional capacity of the patients [25], NDI was used to assess the above and monitor possible significant differences between measurements. The NDI was significantly associated with greater extension maximum and average. This finding is supported by Aimi et al. who found a significant correlation between NDI and extension [25].

In light of these findings, we conclude that the Moover 3D sensor is a good reliable tool for measuring the ACROM as it has shown acceptable intra- and inter-rater values. In contrast to studies suggesting the need for two sensors for higher precision [3,8,9] and elimination of compensating movements of the thoracic segment [3,11,12], the active and passive stabilization that was provided in our study may have reduced this effect. Thus, a single inertial sensor on the forehead could be acceptable and can be used in a clinical setting for the measurement of ACROM in chronic neck pain patients.

Limitations

There are potential limitations in this study. First of all, only chronic neck pain patients were included in this study; thus, the generalization of these findings cannot be extrapolated to other populations. Moreover, slippage of the sensor, because of the elastic bands, could influence the reliability results [10]. The use of the same bands likely reduced this bias. Finally, the subjects’ cognitive skills, habits, activities, and sleep quality as well as the weather and temperature could affect the results of the measurements [3].

## Conclusions

The present study shows that the Moover 3D inertial sensor is a reliable tool for ACROM measurement in chronic neck pain patients. Additionally, the NDI scale showed good intra-rater reliability in the same sample. Both intra-rater and inter-rater reliability of the 3D Moover were shown to be acceptable for a period of 48 hours apart in a Greek urban setting and sample. This inertial sensor could offer a possible use in a clinical setting.
